# Supplementary material for: The dichotomous and incomplete adaptive immunity in COVID-19 patients with different disease severity
Source: Signal Transduct Target Ther. 2021 Mar 8;6:113. doi: 10.1038/s41392-021-00525-3 (PMC7938043; doi:10.1038/s41392-021-00525-3)
Supplement: Supplementary file 1 — supplementary data [file 41392_2021_525_MOESM1_ESM.docx]

Supplementary Materials for

**The dichotomous and incomplete adaptive immunity in COVID-19 patients with different disease severity**

Leiqiong Gao, Jing Zhou, Sen Yang, Lisha Wang, Xiangyu Chen, Yang Yang, Ren Li, Zhiwei Pan, Jing Zhao, Zhirong Li, Qizhao Huang, Jianfang Tang, Li Hu, Pinghuang Liu, Guozhong Zhang ^*^, Yaokai Chen^*^& Lilin Ye^*^

Correspondence to: Guozhong Zhang (zhanggz@cau.edu.cn), Yaokai Chen (yaokaichen@hotmail.com) and Lilin Ye (yelilinlcmv@tmmu.edu.cn).

**This PDF file includes:**

Figures. S1 to S2

Tables S1 to S4

Figure. S1.


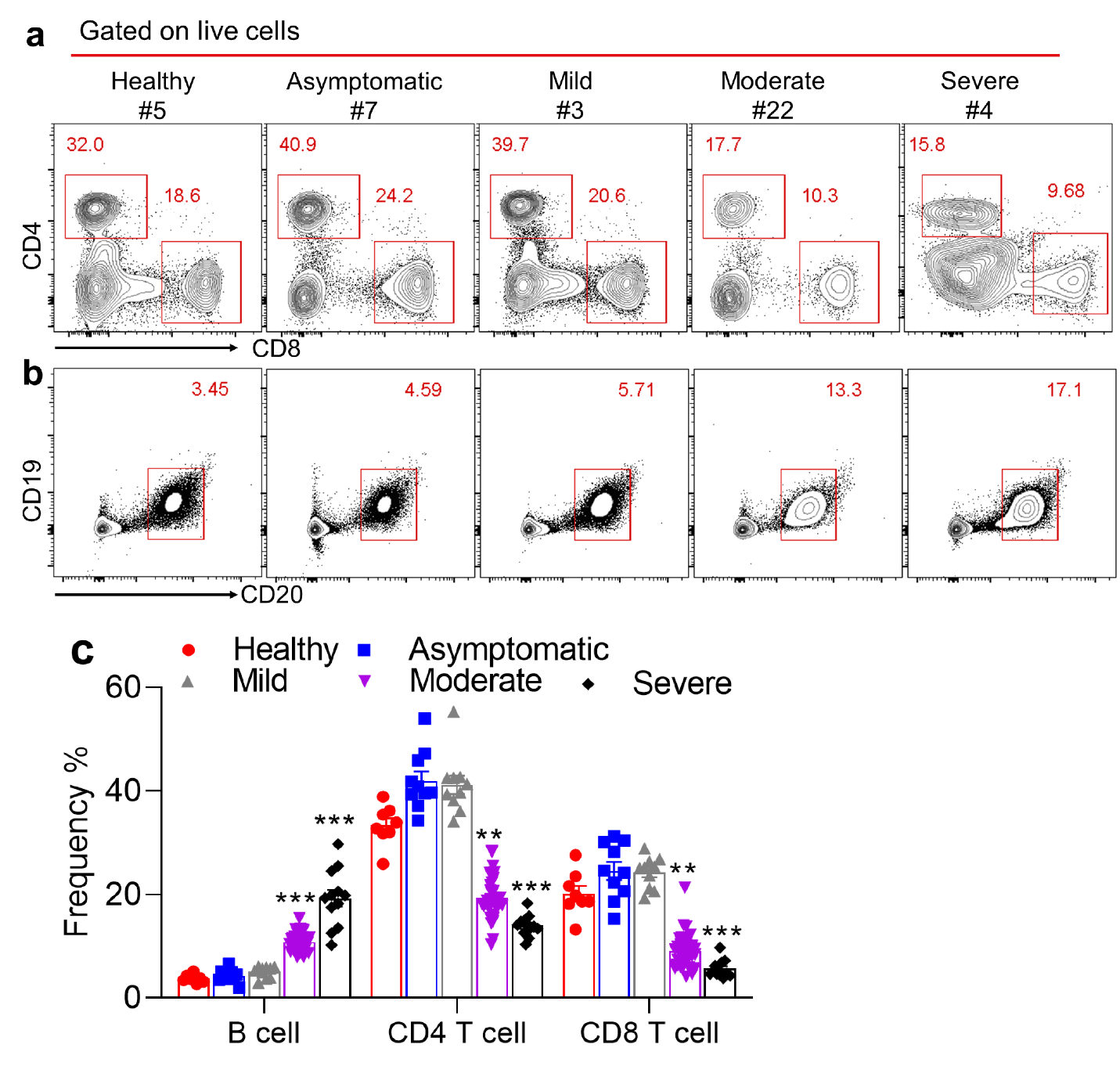


**Figure. S1**, **Frequency of B cells, CD4^+^ T cells and CD8^+^ T cells in total live PBMCs in COVID-19 patients with different severity.**

Samples of **a** and **b** were from Healthy (#5), Asymptomatic (#7), Mild (#3), Moderate (#22), severe (#4).

**a, b,** Representative flow cytometry plots of CD4^+^ and CD8^+^ T cells (**a**) and B cells (**b**) in total live PBMCs in convalescent-phase COVID-19 patients.

**c,** Percentages of CD4^+^ T cells, CD8^+^ T cells an B cells, summarized from (**a, b**), in COVID-19 recovered patients with different disease severity.

Bars represent the mean ± SEM. *P* values were calculated based on Bonferroni of one-way ANOVA analysis. ***, *p*<0.001, and **, *p*<0.01.

Figure. S2.


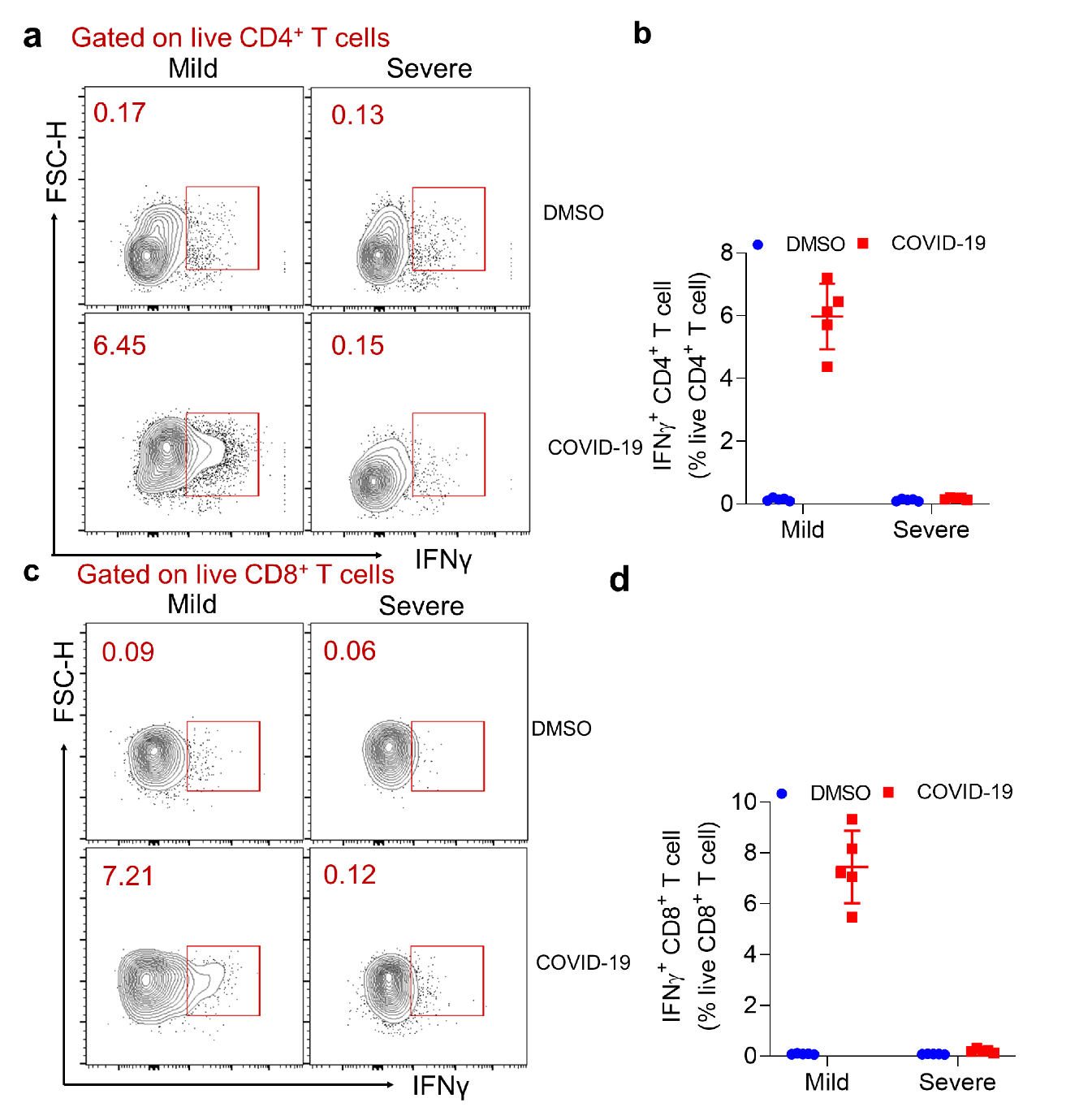


**Figure. S2 Percentage of SARS-CoV2 specific IFNγ^+^CD4^+^ and IFNγ^+^CD8^+^ T cell in COVID-19 mild and severe convalescent patients.**

PBMCs of mild and severe recovered COVID-19 patients (n=5, respectively) were stimulated with SARS-CoV-2 peptides cocktails for 14 hours, Golgi-Plug containing Golgi-stop and DNase were added into cell culture for another 4 hours.

Samples of **a** and **c** were from mild (#8) and severe (#6).

Table S1. Characteristics of individual patients in this study

|  | **Donors** | **Age (years)** | **Gender** | **Duration of hospitalization**  **(days)** | **Onset of discharge to sample collection (days)** | **Hospital admission date (medium IQR)** |
| --- | --- | --- | --- | --- | --- | --- |
| Healthy | **#1** | 35 | Male |  |  |  |
|  | **#2** | 38 | Male |  |  |  |
|  | **#3** | 29 | Male |  |  |  |
|  | **#4** | 26 | Male |  |  |  |
|  | **#5** | 28 | Female |  |  |  |
|  | **#6** | 32 | Female |  |  |  |
|  | **#7** | 34 | Female |  |  |  |
|  | **#8** | 25 | Female |  |  |  |
| **Asymptomatic** | **#1** | 19 | Male | 27 | 11 | 2020/3/22 |
|  | **#2** | 22 | Female | 12 | 12 | 2020/4/07 |
|  | **#3** | 23 | Male | 20 | 14 | 2020/3/28 |
|  | **#4** | 20 | Female | 24 | 15 | 2020/4/09 |
|  | **#5** | 19 | Male | 39 | 10 | 2020/4/09 |
|  | **#6** | 26 | Female | 30 | 14 | 2020/3/25 |
|  | **#7** | 20 | Male | 24 | 14 | 2020/3/26 |
|  | **#8** | 40 | Male | 51 | 14 | 2020/3/22 |
|  | **#9** | 56 | Female | 23 | 13 | 2020/3/28 |
|  | **#10** | 19 | Male | 20 | 12 | 2020/4/15 |
| **Mild** | **#1** | 19 | Male | 27 | 10 | 2020/4/06 |
|  | **#2** | 23 | Female | 26 | 11 | 2020/4/11 |
|  | **#3** | 20 | Female | 31 | 11 | 2020/4/08 |
|  | **#4** | 26 | Female | 16 | 13 | 2020/4/23 |
|  | **#5** | 32 | Female | 20 | 14 | 2020/4/21 |
|  | **#6** | 56 | Female | 28 | 14 | 2020/4/24 |
|  | **#7** | 28 | Male | 13 | 12 | 2020/4/22 |
|  | **#8** | 33 | Male | 12 | 14 | 2020/2/08 |
|  | **#9** | 42 | Female | 24 | 14 | 2020/2/17 |
|  | **#10** | 33 | Male | 18 | 14 | 2020/2/07 |
| **Moderate** | **#1** | 33 | Male | 10 | 14 | 2020/2/09 |
|  | **#2** | 50 | Female | 9 | 14 | 2020/2/03 |
|  | **#3** | 62 | Male | 10 | 14 | 2020/2/02 |
|  | **#4** | 35 | Male | 12 | 14 | 2020/2/27 |
|  | **#5** | 54 | Female | 16 | 14 | 2020/3/02 |
|  | **#6** | 42 | Female | 21 | 10 | 2020/2/15 |
|  | **#7** | 41 | Female | 24 | 14 | 2020/2/13 |
|  | **#8** | 43 | Female | 29 | 14 | 2020/2/14 |
|  | **#9** | 34 | Female | 14 | 12 | 2020/3/04 |
|  | **#10** | 63 | Female | 18 | 12 | 2020/2/16 |
|  | **#11** | 64 | Male | 19 | 11 | 2020/2/04 |
|  | **#12** | 60 | Female | 17 | 13 | 2020/3/06 |
|  | **#13** | 24 | Female | 10 | 14 | 2020/3/03 |
|  | **#14** | 20 | Female | 11 | 12 | 2020/2/03 |
|  | **#15** | 40 | Male | 8 | 10 | 2020/2/13 |
|  | **#16** | 39 | Male | 11 | 10 | 2020/2/14 |
|  | **#17** | 55 | Female | 24 | 12 | 2020/3/04 |
|  | **#18** | 63 | Female | 20 | 11 | 2020/2/16 |
|  | **#19** | 37 | Male | 13 | 12 | 2020/2/04 |
|  | **#20** | 35 | Female | 25 | 14 | 2020/3/06 |
|  | **#21** | 46 | Female | 15 | 14 | 2020/3/03 |
|  | **#22** | 29 | Male | 18 | 14 | 2020/2/03 |
|  | **#23** | 26 | Male | 9 | 14 | 2020/2/13 |
|  | **#24** | 20 | Female | 27 | 14 | 2020/2/03 |
|  | **#25** | 55 | Male | 19 | 14 | 2020/2/13 |
|  | **#26** | 67 | Male | 13 | 13 | 2020/2/10 |
|  | **#27** | 31 | Male | 7 | 10 | 2020/2/05 |
|  | **#28** | 33 | Male | 18 | 11 | 2020/2/14 |
|  | **#29** | 38 | Male | 26 | 10 | 2020/2/18 |
|  | **#30** | 30 | Male | 10 | 14 | 2020/2/06 |
|  | **#31** | 38 | Female | 27 | 13 | 2020/2/13 |
|  | **#32** | 65 | Female | 17 | 16 | 2020/2/10 |
| **Severe** | **#1** | 72 | Male | 29 | 17 | 2020/2/06 |
|  | **#2** | 65 | Female | 34 | 16 | 2020/2/23 |
|  | **#3** | 74 | Female | 16 | 14 | 2020/2/07 |
|  | **#4** | 64 | Male | 27 | 14 | 2020/2/14 |
|  | **#5** | 58 | Male | 20 | 14 | 2020/3/12 |
|  | **#6** | 65 | Male | 13 | 14 | 2020/3/05 |
|  | **#7** | 43 | Female | 17 | 14 | 2020/2/21 |
|  | **#8** | 49 | Female | 31 | 17 | 2020/2/16 |
|  | **#9** | 54 | Female | 26 | 15 | 2020/2/18 |
|  | **#10** | 67 | Female | 28 | 14 | 2020/3/13 |
|  | **#11** | 72 | Male | 29 | 15 | 2020/3/07 |
|  | **#12** | 69 | Male | 22 | 14 | 2020/2/24 |

Table S2. Summary of P values in Figure1-4

1. ***P* values of Figure 1c**

|  | Healthy | Asymptomatic | Mild | Moderate | Severe |
| --- | --- | --- | --- | --- | --- |
| Healthy | / | * | ** | *** | *** |
| Asymptomatic | / | / | * | *** | *** |
| Mild | / | / | / | ** | *** |
| Moderate | / | / | / | / | ** |

1. ***P* values of Figure 1d**

|  | Healthy | Asymptomatic | Mild | Moderate | Severe |
| --- | --- | --- | --- | --- | --- |
| Healthy | / | ns (>0.99) | ns (>0.99) | *** | *** |
| Asymptomatic | / | / | ns (>0.99) | *** | *** |
| Mild | / | / | / | *** | *** |
| Moderate | / | / | / | / | ** |

1. ***P* values of Figure 1e**

|  | Healthy | Asymptomatic | Mild | Moderate | Severe |
| --- | --- | --- | --- | --- | --- |
| Healthy | / | ns (>0.99) | ns (0.37) | *** | *** |
| Asymptomatic | / | / | ns (>0.99) | *** | *** |
| Mild | / | / | / | ** | *** |
| Moderate | / | / | / | / | ** |

1. ***P* values of Figure 1f**

|  | Healthy | Asymptomatic | Mild | Moderate | Severe |
| --- | --- | --- | --- | --- | --- |
| Healthy | / | ns (>0.99) | ns (>0.99) | *** | *** |
| Asymptomatic | / | / | ns (>0.99) | *** | *** |
| Mild | / | / | / | ** | *** |
| Moderate | / | / | / | / | *** |

1. ***P* values of Figure 1h**

|  | Healthy | Asymptomatic | Mild | Moderate | Severe |
| --- | --- | --- | --- | --- | --- |
| Healthy | / | ns (>0.99) | ns (>0.99) | *** | *** |
| Asymptomatic | / | / | ns (>0.99) | ** | *** |
| Mild | / | / | / | ** | *** |
| Moderate | / | / | / | / | *** |

1. ***P* values of Figure 1i**

|  | Healthy | Asymptomatic | Mild | Moderate | Severe |
| --- | --- | --- | --- | --- | --- |
| Healthy | / | ns (>0.99) | ns (0.28) | ** | *** |
| Asymptomatic | / | / | ns (0.71) | ** | *** |
| Mild | / | / | / | * | ** |
| Moderate | / | / | / | / | * |

1. ***P* values of Figure 2c**

|  | Healthy | Early | Middle | Convalescence |
| --- | --- | --- | --- | --- |
| Healthy | / | *** | *** | * |
| Early | / | / | ns (0.24) | * |
| Middle | / | / | / | ns (0.051) |

1. ***P* values of Figure 2d**

|  | Healthy | Early | Middle | Convalescence |
| --- | --- | --- | --- | --- |
| Healthy | / | *** | *** | ns (>0.99) |
| Early | / | / | ns (0.07) | ** |
| Middle | / | / | / | * |

1. ***P* values of Figure 2e**

**S1**

|  | Healthy | Early | Middle | Convalescence |
| --- | --- | --- | --- | --- |
| Healthy | / | ns (0.41) | * | ns (>0.99) |
| Early | / | / | ns (>0.99) | ns (0.73) |
| Middle | / | / | / | * |

**S2**

|  | Healthy | Early | Middle | Convalescence |
| --- | --- | --- | --- | --- |
| Healthy | / | ** | *** | ns (>0.99) |
| Early | / | / | ns (0.07) | ns (0.052) |
| Middle | / | / | / | ** |

1. ***P* values of Figure 2g**

|  | Healthy | Early | Middle | Convalescence |
| --- | --- | --- | --- | --- |
| Healthy | / | * | * | ns (>0.99) |
| Early | / | / | ns (>0.99) | ns (0.053) |
| Middle | / | / | / | ns (0.11) |

1. ***P* values of Figure 2h**

|  | Healthy | Early | Middle | Convalescence |
| --- | --- | --- | --- | --- |
| Healthy | / | * | * | ns (>0.99) |
| Early | / | / | ns (0.67) | ns (0.22) |
| Middle | / | / | / | ns (>0.99) |

1. ***P* values of Figure 3b.**

|  | Healthy | Asymptomatic | Mild | Moderate | Severe |
| --- | --- | --- | --- | --- | --- |
| Healthy | / | *** | *** | * | ns (0.57) |
| Asymptomatic | / | / | ns (>0.99) | *** | *** |
| Mild | / | / | / | ** | *** |
| Moderate | / | / | / | / | ns (>0.99) |

1. ***P* values of Figure 3d.**

|  | Healthy | Asymptomatic | Mild | Moderate | Severe |
| --- | --- | --- | --- | --- | --- |
| Healthy | / | *** | *** | ns (>0.99) | ns (>0.99) |
| Asymptomatic | / | / | ns (>0.99) | *** | *** |
| Mild | / | / | / | *** | *** |
| Moderate | / | / | / | / | ns (>0.99) |

1. ***P* values of Figure 3f.**

|  | Healthy | Asymptomatic | Mild | Moderate | Severe |
| --- | --- | --- | --- | --- | --- |
| Healthy | / | *** | *** | ** | ns (0.74) |
| Asymptomatic | / | / | ns (>0.99) | ** | *** |
| Mild | / | / | / | ** | *** |
| Moderate | / | / | / | / | ns (0.96) |

1. ***P* values of Figure 3h.**

|  | Healthy | Asymptomatic | Mild | Moderate | Severe |
| --- | --- | --- | --- | --- | --- |
| Healthy | / | *** | *** | * | ns (>0.99) |
| Asymptomatic | / | / | ns (>0.99) | *** | *** |
| Mild | / | / | / | *** | *** |
| Moderate | / | / | / | / | ns (0.65) |

1. ***P* values of Figure 4b.**

|  | Healthy | Early | Middle | Convalescence |
| --- | --- | --- | --- | --- |
| Healthy | / | *** | *** | *** |
| Early | / | / | ns (>0.99) | ns (>0.99) |
| Middle | / | / | / | ns (>0.99) |

1. ***P* values of Figure 4d.**

|  | Healthy | Early | Middle | Convalescence |
| --- | --- | --- | --- | --- |
| Healthy | / | *** | *** | *** |
| Early | / | / | ns (>0.99) | ns (>0.99) |
| Middle | / | / | / | ns (>0.99) |

1. ***P* values of Figure 4f.**

|  | Healthy | Early | Middle | Convalescence |
| --- | --- | --- | --- | --- |
| Healthy | / | *** | *** | *** |
| Early | / | / | ns (>0.99) | ns (>0.99) |
| Middle | / | / | / | ns (>0.99) |

1. ***P* values of Figure 4h.**

|  | Healthy | Early | Middle | Convalescence |
| --- | --- | --- | --- | --- |
| Healthy | / | *** | *** | *** |
| Early | / | / | ns (>0.99) | ns (>0.99) |
| Middle | / | / | / | ns (>0.99) |

*P* values were calculated based on Bonferroni of one-way analysis. ***, *p*<0.0001, **, *p*<0.001, and *, *p*<0.05.

Table S3. SARS-CoV-2 T cell epitopes

| T cell phenotype | Amino acid residue SARS-CoV-2 Amino acid sequence |
| --- | --- |
| CD4/CD8 | GMEVTPSGTWLTYTGAIKLD |
| CD4 | QALNTLVKQLSSFGAI |
| CD4 | MAYRFNGIGVTQNVLY |
| CD4 | SFLEDLLFNKUTLAD |
| CD8 | LAKDTTEAF |
| CD8 | VLLSVLQQL |
| CD8 | FEYVSQPFI |
| CD8 | QPTESIVRF |

Table S4. Flow cytometry antibodies in this study

| **Antigen** | **Fluorochrome** | **Clone** | **Manufacturer** | **Volume/test (μL)** |
| --- | --- | --- | --- | --- |
| CD8 | FITC | RPA-T8 | Biolegend | 0.125 |
| PD1 | PE | EH12.2H7 | Biolegend | 0.5 |
| CXCR5 |  |  | BD | 1 |
| Biotin |  |  | BD | 0.25 |
| Avidin | PE-Cy7 |  | Biolegend | 0.125 |
| CD45RA | APC | HI100 | Biolegend | 0.125 |
| LIVE/DEAD | APC-Cy7 |  | Thermo fisher | 0.25 |
| CD19 | APC-Cy7 | H1B19 | Biolegend | 0.125 |
| CD4 | BV510 | OKT4 | Biolegend | 0.125 |
| CD20 | FITC | 2H7 | Biolegend | 0.125 |
| CD27 | PE | M-T271 | Biolegend | 0.5 |
| CD19 | PerCP | H1B19 | BD | 1 |
| S1+S2 |  |  | Sino Biological | 1 |
| Avidin | PE-Cy7 |  | Biolegend | 0.125 |
| LIVE/DEAD | APC-Cy7 |  | Thermo fisher | 0.25 |
| IgD | BV510 | IA6-2 | Biolegend | 0.125 |
| TNFα | FITC | MAb11 | Biolegend | 0.5 |
| Perforin | PE | B-D48 | Biolegend | 0.5 |
| CD8 | PerCP | RPA-T8 | Biolegend | 0.125 |
| GZMB | APC | GB11 | Biolegend | 0.5 |
| LIVE/DEAD | APC-Cy7 |  | Thermo fisher | 0.25 |
| IFNγ | BV421 | 4S. B3 | Biolegend | 0.5 |
| CD4 | BV510 | OKT4 | Biolegend | 0.125 |
